# A straightforward method to quantify circulating mRNAs as biomarkers of colorectal cancer

**DOI:** 10.1038/s41598-023-29948-4

**Published:** 2023-02-15

**Authors:** Marie Grosgeorges, Laurence Picque Lasorsa, Brice Pastor, Corinne Prévostel, Evelyne Crapez, Cynthia Sanchez, Florence Frayssinoux, Marta Jarlier, Véronique Pezzella, Laure Monard, Marc Ychou, Alain R. Thierry, Thibault Mazard, Philippe Blache

**Affiliations:** 1 Institut de Recherche en Cancérologie de Montpellier INSERM U1194, Univ Montpellier, Inserm, ICM, CNRS, Campus Val d’Aurelle 208 avenue des Apothicaires, 34298 Montpellier cedex 5, France; 2 UNICANCER R&D, Paris, France

**Keywords:** Diagnostic markers, Tumour biomarkers

## Abstract

Optimizing the biomarker combination to be analyzed in liquid biopsies should improve personalized medicine. We developed a method to purify circulating cell-free mRNAs from plasma samples and to quantify them by RT-qPCR. We selected three candidate colorectal cancer biomarkers (*B2M*, *TIMP-1*, and *CLU*). Their mRNA levels were significantly higher in plasma of patients with metastatic colorectal cancer patients (mCRC) (n = 107) than in healthy individuals (HI) (n = 53). To increase the discriminating performance of our method, we analyzed the sum of the three mRNA levels (BTC index). The area under the ROC curve (AUC) to estimate the BTC index capacity to discriminate between mCRC and HI plasma was 0.903. We also determined the optimal BTC index cut-off to distinguish between plasma samples, with 82% of sensitivity and 93% of specificity. By using mRNA as a novel liquid biopsy analytical parameter, our method has the potential to facilitate rapid screening of CRCm.

## Introduction

Cancer detection and prognostic classification as well as treatment response monitoring need to be improved. For instance, the diagnosis of most cancers is still based on histological examination of biopsy samples. However, biopsies are an invasive, expensive and time-consuming method. To overcome these disadvantages, the analysis of circulating nucleic acids as non-invasive biomarkers has generated great interest^1^. Cell-free DNA (cfDNA) and RNA (mRNA and microRNA) are released by cells and their analysis/quantification in blood can bring valuable information. Many studies have shown the clinical interest of circulating DNA analysis for cancer management^2^. Nevertheless, a large number of biomarkers need to be identified and/or characterized to move towards a more personalized medicine. For instance, the first study showing the clinical value of the detection of tyrosinase mRNA in serum of patients with metastatic melanoma dates from 1999^3^. However, the use of this mRNA as a biomarker in liquid biopsy is facing difficulties due to its instability and low abundance, and this might explain why few studies have been published so far.

Moreover, the analysis of circulating nucleic acids by whole-transcriptome sequencing is expensive and this method is available only in few centers. Therefore, we developed an efficient, rapid, and cost-effective RT-qPCR-based method to purify and analyze mRNAs from small volumes of blood plasma. Using this method, we could show that the plasma concentration of some mRNAs was significantly higher in plasma samples from patients with metastatic colorectal cancer (mCRC) than in healthy individuals (HI). These results may provide new perspectives for the management of colorectal cancer.

## Results

### B2M, TIMP-1 and CLU mRNA levels in patients with metastatic colorectal cancer

**Figure 1:**
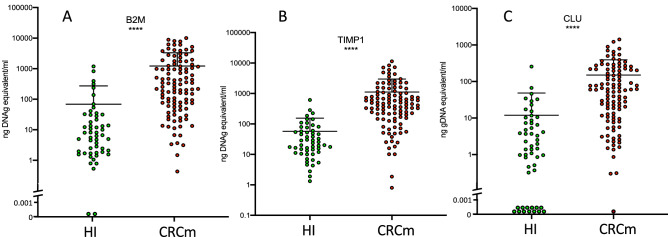
*B2M*, *TIMP-1* and *CLU* mRNA levels are increased in the plasma of patients with metastatic colorectal cancer. *B2M* (**A**), *TIMP-1* (**B**) and *CLU* (**C**) mRNA levels in plasma samples from HI (n = 53) and patients with mCRC (n = 107). Each dot represents one sample, and the mean ± SD for each group is indicated. In the logarithmic scale, values = 0 could not be plotted, log axis was broken and the points at “0” are shown. ****p < 0.0001 (non-parametric Wilcoxon–Mann–Whitney test).

**Figure 2:**
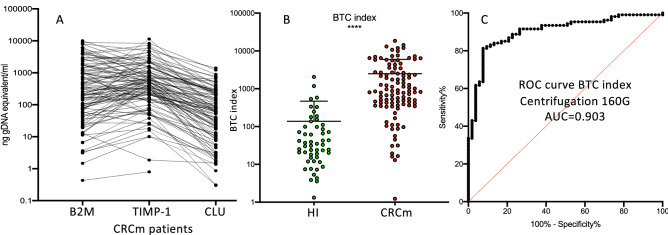
The BTC index as a candidate biomarker of metastatic CRC. (**A**) The mRNA levels of *B2M*, *TIMP-1* and *CLU* were plotted for each patient and were connected by a line; values = 0 could not be plotted on the logarithmic scale. (**B**) Dot plot showing the BTC index (i.e. the sum of the *B2M*, *TIMP-1,* and *CLU* mRNA levels) in HI (n = 53) and in patients with mCRC (n = 107); each dot represents one sample and the mean ± SD is indicated for each group. (**C**) Receiver operating characteristic curve showing that the BTC index can discriminate between serum samples from patients with mCRC (n = 107) and HI (n = 53). ****p < 0.0001 (non-parametric Wilcoxon–Mann–Whitney test).

**Figure 3:**
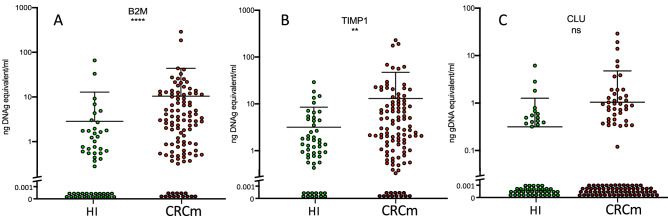
A second centrifugation of the plasma at 16000 g is less efficient to quantify the mRNA. *B2M* (**A**), *TIMP-1* (**B**) and *CLU* (**C**) mRNA levels in plasma samples from HI (n = 53) and patients with mCRC (n = 107). Each dot represents one sample, and the mean and SD for each group is indicated. In the logarithmic scale, values = 0 could not be plotted, log axis was broken and the points at “0” are shown. ****p < 0.0001, ***p < 0.001 (non-parametric Wilcoxon–Mann–Whitney test).

Since our patient population consists of CRCm patients, we selected a number of genes shown to be overexpressed in tumors of colorectal cancer patients based on data from the literature (Table 1). Using our method, we tested all 17 mRNA and we detected only three of them in the plasma of HI: beta2-microglobulin (*B2M*), tissue inhibitor of metalloproteinases 1 (*TIMP-1*), and clusterin (*CLU*) (Fig. 1). After confirming that all three biomarkers could be detected in HI plasma samples, their plasma levels were compared in patients with mCRC (n = 107) and in HI (n = 53). *B2M*, *TIMP-1* and *CLU* mRNA levels were significantly higher in plasma samples of patients with mCRC than in HI (Table 2, Fig. 1). Plotting the plasma level of the three mRNAs for each patient (Fig. 2A) highlighted that their profiles were specific to each patient. When plasmas underwent a second centrifugation at 16,000 × g (instead of the optimal 160 × g), the mRNA level of each mRNA was lower (Fig. 3). If the plasma has already undergone a second centrifugation at 16000 g, as for plasmas prepared for circulating DNA analysis, only B2M and TIMP-1 measurement can give informative results (Fig. 3B,C).

**Table 1:** List of mRNA tested in plasma.

Name	Genebank	Primers	Detection in plasma
*VCAM1* : vascular cell adhesion molecule 1	NM_001078.2	F : AAAAGCGGAGACAGGAGACA R : ATCCTTCAACTGGGCCTTTC	NO
*LGALS3* : galectin 3	NM_002306.4	F : TCCACTTTAACCCACGCTTC R : CTTTCTTCCCTTCCCCAGTT	NO
*SERPINE1* : serpin family E member 1	NM_001386460.1	F: AGGCAGCTCGGATTCAACTA R: TCTTTGTGCCCTACCCTCTG	NO
*CRP* : C-reactive protein	NM_001329057.2	F: TCAAAGCCTTCACTGTGTGC R: TGTCTCTTGGTGGCATACGA	NO
*CLU* : Clusterin	NM_001831	F: GAGACCAGGGAATCAGAGACA R: TTTCAGGCAGGGCTTACACT	YES
*ANG* : Angiogenin	NM_001145.4	F: CATCTTTGCGTTTTCTACCG R: TTGTTTCTCACCCGCTTCTT	NO
*COL6A3* : collagen type VI alpha 3 chain	NM_004369.4	F: CAGCAAGGATGAAGTGCAGA R: GACACGTACTCCAGGGCATT	NO
*TIMP1* : TIMP metallopeptidase inhibitor 1	NM_003254.3	F: CCGGAGTGGAAGCTGAAG R: TTGTCCGGAAGAAAGATGGG	YES
*TFRC* : transferrin receptor	NM_001128148.3	F : GAAAAGAGGGGACCAGAAGC R : TATGGGGGAAGGGACAGAG	NO
*B2M*: beta-2-microglobulin	NM_004048.4	F: TTCTACTTTGAGTGCTGTCTCCA R: TTCTCTGCTCCCCACCTCTA	YES
*SPON2* : spondin 2	NM_012445.4	F: GGGACCAAGAGCAGGACTC R: TATCAGGGACGCACTCAGC	NO
*KRT19* : keratin 19	NM_002276.5	F:CTGGGTAGAGGGATGGGAAG R: ATTGGCAGGTCAGGAGAAGA	NO
*S100A9* : S100 calcium binding protein A9	NM_002965.4	F: TGAAAAGGTCATAGAACACATCA R: TAGCCTCGCCATCAGCAT	NO
*CEACAM5* : CEA cell adhesion molecule 5	NM_004363.6	F : TGTCGGCATCATGATTGGAGT R : TGAAGAAACTACACCAGGGCT	NO
*MMP8* : matrix metallopeptidase 8	NM_002424.3	F : AAAGAAAGCCAGGAGGGGTA R : TGGAGTAAGAGCAGAAATGGAA	NO
*MMP9* : matrix metallopeptidase 9	NM_004994	F : GTGCCATGTAAATCCCCACT R : TCCCTTTCCTCCAGAACAGA	NO
*MACC1* : MET transcriptional regulator MACC1	NM_182762.4	F: TCCAACCCCAAACCTAAAAA R: TTGATTTCCTCCTTCTTCTGC	NO

**Table 2:** *B2M*. *TIMP-1* and *CLU* mRNA levels in plasma samples from HI and patients with mCRC.

	HI (n = 53)	mCRC (n = 103)	*P* value
B2M			< 0.0001****
Median	6.650	257.9	
Range	0–1184	0.43–10,044	
Mean	68.48	1223	
SEM	28.1	200.2	
SD	204.6	2071	
TIMP-1			< 0.0001****
Median	23.37	467.1	
Range	1.32–612.9	0.8–11,360	
Mean	57.45	1117	
SEM	13.32	179.5	
SD	97	1856	
CLU			< 0.0001****
Median	1.7	59.10	
Range	0–256	0–1416	
Mean	11.91	150	
SEM	5	23.26	
SD	36.56	240.6	
BTC index			< 0.0001****
Median	33	867	
Range	1.3–2053	1.2–18,445	
Mean	138	2491	
SEM	13,919	107,011	
SD	331	3501	

All values are in ng of genomic DNA equivalents per ml. *P* values were calculated with the non-parametric Wilcoxon–Mann–Whitney test.

### An index (BTC) that combines the three mRNAs (B2M, TIMP-1, and CLU) as a marker of mCRC

The BTC index (i.e. the sum of the plasma levels of the three mRNAs for each sample) also was significantly higher in patients with mCRC than in HI (Fig. 2B and Table 2). This suggested that the BTC index could be used to summarize all results. The AUC-ROC analysis showed that the capacity of the BTC index to discriminate between mCRC and HI plasma samples was higher than that of each mRNA (Fig. 2C and Table 3). The BTC index cut-off of 340, determined with the Youden’s index, discriminated between mCRC and HI plasma samples with a sensitivity of 82% and a specificity of 91%.

**Table 3:** ROC analysis.

B2M	
AUC	0.876
Std. Error	0.029
95% CI	0.818–0.933
TIMP-1	
AUC	0.899
Std. Error	0.024
95% CI	0.851–0.947
CLU	
AUC	0.860
Std. Error	0.03
95% CI	0.802–0.919
BTC index	
AUC	0.903
Std. Error	0.025
95% CI	0.854–0.952

### Effect of the time between blood collection and plasma preparation, gender and age on the BTC index

**Figure 4:**
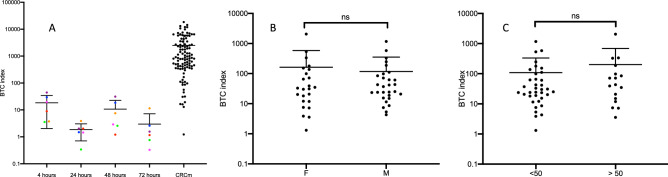
Influence of the time between blood collection and plasma preparation, gender and age on the BTC index. (**A**) 6 HI blood samples were separated into 4 aliquots and the plasmas were prepared 4, 24, 48 and 72 h later (one spot color per individual). (**B**) The BTC index is not different between women (F) and men (M) (healthy donors). (**C**) The BTC index is not different between healthy individuals younger or older than 50 years.

Finally, mRNA quantification (i.e. BTC index) was not influenced by the time between blood collection and plasma preparation (Fig. 4A), donor sex (Fig. 4B), and donor age (Fig. 4C). 6 HI blood samples were separated into 4 aliquots and the plasmas were prepared 4, 24, 48 and 72 h later (one spot color per individual) (Fig. 4A). Small variations in BTC index were observed which nevertheless cannot question the difference in BTC index between HI and patients. (The BTC index is not different between women (F) and men (M) (healthy donors) (Fig. 4B). The BTC index is not different between healthy individuals younger or older than 50 years (Fig. 4C). 50 years is the age at which the risk of developing colorectal cancer increases significantly and at which screening programs are initiated.

## Discussion

A recent study showed that analysis of the circulating transcriptome in patients with cancer has a great potential for cancer detection^4^. However, this study was performed by sequencing large plasma volumes (8 ml), which in clinical routine, reduces the possibility of performing multiple analyses in parallel. Therefore, our goal was to develop a simple-to-use, non-invasive, and inexpensive method that requires very few biological resources.

Our RT-PCR-based method for circulating mRNA quantification has many advantages:

1. Blood samples from centers distant from the place where the analysis will be performed can be used because mRNAs are stable for few days when collected in Streck Cell-Free DNA BCT® tubes;
2. As all analyses can be performed using frozen plasma samples, retrospective studies can be easily carried out and sample analysis can be performed in batches;
3. A very small volume of plasma (200 µl) is sufficient for circulating mRNA analysis, which allows performing many other analyses in parallel;
4. Thanks to the absolute quantification by qPCR using gDNA, standardizing the analysis is easy.

Our study was performed using plasma samples that were centrifuged at 160 × g before freezing, according to the optimal pre-analytical conditions we determined before this study. Indeed, the use of plasma samples that were frozen after centrifugation at 16,000 × g reduced the sensitivity of our assays (Fig. 3).

The three mRNAs we selected and studied are of interest in the context of colorectal cancer for the following reasons. B2M is a component of the human leukocyte antigen class I complex that is involved in the presentation of antigenic peptides on the surface to CD8 + cytotoxic T cells, and thus participates in a critical step of the cell-mediated antitumor immune response^5^. *B2M* overexpression is associated with poor survival in patients with stage I, II and III CRC^6^, and elevated serum B2M protein levels are associated with higher risk of CRC^7^. Matrix metalloproteinases and TIMPs are involved in extracellular matrix degradation, which is an essential element of tumor cell invasion. Particularly, TIMP-1 stimulates cell proliferation^8^, promotes tumor invasion and metastasis development^9^, inhibits apoptosis^10^, and induces angiogenesis^11^. TIMP-1 is a potential prognostic indicator in CRC and plays an important role in promoting CRC tumorigenesis and metastasis^12–15^. CLU is a pleiotropic protein with a wide range of functions, such as membrane recycling, tissue remodeling, cell–cell and cell-substrate interactions, stabilization of stressed proteins, apoptosis inhibition^16^. In CRC, high *CLU* mRNA level in tumors predicts poor prognosis^17^, and CLU protein level is increased in the serum of patients with CRC^18^. Therefore, the three mRNAs we chose to study are good indicators of the disease.

Here we show that the level of the three selected mRNAs (*B2M*, *TIMP-1* and *CLU*) was much higher in plasma samples of patients with mCRC, and the BTC index was very sensitive and specific. As this work was carried using plasma samples obtained in Streck Cell-Free DNA BCT® tubes from an ongoing clinical trial in patients with mCRC, we could only analyze patients with mCRC. In future works, we would like to test our method also in patients with CRC at different stages to determine whether it could be used for early CRC detection and/or for monitoring the disease course/response to treatment. Moreover, we would like to determine whether this method could be used also in patients with other cancer types after selection of biomarkers specific for each cancer.

Our data also raise the question of the mRNA origin: extracted from intact cells in the plasma samples, mRNA released by circulating cells, or mRNA released by tissues and/or tumor cells. We think unlikely the presence of intact cells in the tested plasma samples after two centrifugation steps. However, we cannot exclude the possibility that some of these mRNAs originated from residual platelets, particularly from tumor-educated platelets (TEP)^19–21^. It should be noted that *CLU* mRNA level is decreased in platelets of patients with CRC, even at early stages^22^. This weakens the hypothesis that the tested mRNAs could originate from residual platelets.

We do not claim that our finding may replace of even compete with existing liquid biopsy analysis such as circulating DNA, circulating tumor cells or others. Our intention is to provide circulating mRNAs as an additional assay that could strengthen the global performances of liquid biopsy for clinical applications. For example, a high circulating DNA level even if associated with a mutation does not certify that the patient has a tumor, and further not an intestinal tumor. To the same extent, if circulating tumor cells are detected in blood samples, this does not provide any information on the tumor location.

To conclude, our method can be used for the rapid and efficient screening of plasma samples from patients with mCRC using mRNA as a novel analytical parameter in liquid biopsy.

## Methods

### Blood sample collection

Whole blood samples were collected in Streck Cell-Free DNA BCT® tubes and stored at room temperature before plasma preparation. These tubes are widely used to analyze circulating DNA from blood samples that must be sent to a centralized laboratory and thus may be transported for several days^23,24^. Upon reception, blood was centrifuged at 1200 × g at 4 °C for 10 min to separate plasma. To avoid plasma contamination by blood cells, the first 1 ml of plasma above the buffy coat was not collected. Plasma samples were then centrifuged at 160 × g at 4 °C for 10 min, aliquoted (200 µl/aliquot), and stored for a short time (no more than 3 days) at -20 °C until RNA purification. Longer plasma storage times should be performed at -80 °C.

### RNA isolation, reverse transcription and real-time quantitative PCR (RT-qPCR)

Plasma aliquots (200 µl) were thawed on ice and RNA was prepared and purified using the Plasma/Serum RNA/DNA Purification Mini Kit (Norgen, reference 55,200) according to the manufacturer’s protocol. Total RNA was eluted in 12 µl of elution solution. Reverse transcription was performed with the QuantiTect Reverse Transcription Kit (Qiagen; reference 20,531). Briefly, 2 µl of gDNA wipeout buffer were added to the 12 µl of eluted RNA and incubated at 42 °C for 5 min to eliminate contaminating genomic DNA and reverse transcription was performed in a final volume of 20 µl for 30 min at 42 °C. This was followed by qPCR using the iQ™ SYBR® Green Supermix (BIO-RAD) and a LightCycler® 480 apparatus (Roche). The reaction volume: 1 µl of sample, 1 µl of each primer (final concentration 1.25 µM), 5 µl of SYBR® Green Supermix and 2 µl of H2O. Additional details regarding qPCR setup, specificity and accuracy are provided in Supplementary dataset 1. The absolute quantification was performed with human genomic DNA (gDNA) (Promega, reference G1471) and the results are expressed in ng gDNA equivalent. To confirm that the prepared cDNA was not contaminated by genomic DNA, two primers that hybridize only to genomic DNA and not to RNA-derived cDNA sequences (forward, 5’-CACCCATGATATGCCTTAGC-3’, reverse, 5’-TTCTGCTGAACCCAAGAGC-3’) were used as control in each run. This also validated that RNA purification and the gDNA wipeout treatment are required for optimal mRNA quantification. The sequences of the other primer pairs are indicated in Table1.

### Statistics

Differences between groups (HI and patients with mCRC) were assessed with the non-parametric Wilcoxon-Mann–Whitney test. To evaluate the biomarker capacity to discriminate between HI and mCRC samples, the receiver operating characteristic (ROC) curve and the area under the curve (AUC) were determined for each biomarker. AUC are presented with their 95% confidence intervals (95% CI). The optimal threshold value for the biomarker combination (or for each single biomarker) was determined with the Youden’s index (defined as the sum of the sensitivity and specificity minus 1). All p-values are two-sided and the significance level was set at 0.05. Statistical analyses were performed with the Prism8 software.

### Study approval

Blood samples from patients with mCRC were obtained in the framework of the ongoing PANIRINOX phase II randomized clinical trial that compare the efficacy of FOLFIRINOX + panitumumab versus mFOLFOX6 + panitumumab in patients with mCRC selected on the basis of RAS and B-RAF status in circulating DNA (Protocol n. UC-0110/1608. EudraCT n. 2016-001490-33). All patients signed a written informed consent before inclusion. This trial involves 31 French hospitals/cancer centers and was approved by the Sud Méditerranée IV ethics committee. This cohort study was approved by Unicancer, the sponsor of the PANIRINOX study, and by the Agence Nationale de Sécurité du Médicament et des Produits de Santé and the Comités de Protection des Personnes, according to the French national regulatory requirements. Blood samples from HI were provided by the “Etablissement Français du Sang, the blood transfusion center of Montpellier, France (Agreement EFS-PM n. 21PLER2015-0013). All methods were carried out in accordance with relevant guidelines and regulations.

## Supplementary Information

Supplementary Information.

## Data Availability

The datasets used and/or analyzed during the current study are available from the corresponding author. PB is inventor of a patent filing describing the use of this method for circulating cell-free mRNA analysis.
